# The temporal dynamics of *Plasmodium* species infection after artemisinin-based combination therapy (ACT) among asymptomatic children in the Hohoe municipality, Ghana

**DOI:** 10.1186/s12936-023-04712-1

**Published:** 2023-09-14

**Authors:** Felix Ansah, Kwamina Nyame, Rukaya Laryea, Richard Owusu, Denick Amon, Mark-Jefferson Buer Boyetey, Dzidzor Ayeke, Nasibatu Razak, Victor E. Kornu, Sarah Ashitei, Caleb Owusu-Appiah, Jersley D. Chirawurah, James Abugri, Yaw Aniweh, Nicholas Opoku, Colin J. Sutherland, Fred N. Binka, Margaret Kweku, Gordon A. Awandare, Bismarck Dinko

**Affiliations:** 1 West African Centre for Cell Biology of Infectious Pathogens (WACCBIP), College of Basic and Applied Sciences, University of Ghana, Legon, Accra, Ghana; 2 Department of Biochemistry, Cell and Molecular Biology, College of Basic and Applied Sciences, University of Ghana, Legon, Accra, Ghana; 3 Department of Epidemiology and Biostatistics, Fred Newton Binka School of Public Health, University of Health and Allied Sciences, Hohoe, Ghana; 4 Department of Biochemistry and Forensic Sciences, School of Chemical and Biochemical Sciences, C. K. Tedam University of Technology and Applied Sciences, Navrongo, Ghana; 5 Department of Infection Biology, Faculty of Infectious and Tropical Diseases, London School of Hygiene and Tropical Medicine, London, UK; 6 Department of Biomedical Sciences, School of Basic and Biomedical Sciences, University of Health and Allied Sciences, Ho, Ghana; 7 Present Address: Department of Clinical Microbiology, School of Medicine and Dentistry College of Health Sciences, Kwame Nkrumah University of Science and Technology, Kumasi, Ashanti Region Ghana

**Keywords:** Asymptomatic malaria, *Plasmodium* species, Prevalence rate, Artemisinin-based combination therapy (ACT), Parasite clearance

## Abstract

**Background:**

The routine surveillance of asymptomatic malaria using nucleic acid-based amplification tests is essential in obtaining reliable data that would inform malaria policy formulation and the implementation of appropriate control measures.

**Methods:**

In this study, the prevalence rate and the dynamics of *Plasmodium* species among asymptomatic children (n = 1697) under 5 years from 30 communities within the Hohoe municipality in Ghana were determined.

**Results and discussion:**

The observed prevalence of *Plasmodium* parasite infection by polymerase chain reaction (PCR) was 33.6% (571/1697), which was significantly higher compared to that obtained by microscopy [26.6% (451/1697)] (*P* < 0.0001). Based on species-specific analysis by nested PCR, *Plasmodium falciparum* infection [33.6% (570/1697)] was dominant, with *Plasmodium malariae*, *Plasmodium ovale* and *Plasmodium vivax* infections accounting for 0.1% (1/1697), 0.0% (0/1697), and 0.0% (0/1697), respectively. The prevalence of *P. falciparum* infection among the 30 communities ranged from 0.0 to 82.5%. Following artesunate-amodiaquine (AS + AQ, 25 mg/kg) treatment of a sub-population of the participants (n = 184), there was a substantial reduction in *Plasmodium* parasite prevalence by 100% and 79.2% on day 7 based on microscopy and nested PCR analysis, respectively. However, there was an increase in parasite prevalence from day 14 to day 42, with a subsequent decline on day 70 by both microscopy and nested PCR. For parasite clearance rate analysis, we found a significant proportion of the participants harbouring residual *Plasmodium* parasites or parasite genomic DNA on day 1 [65.0% (13/20)], day 2 [65.0% (13/20)] and day 3 [60.0% (12/20)] after initiating treatment. Of note, gametocyte carriage among participants was low before and after treatment.

**Conclusion:**

Taken together, the results indicate that a significant number of individuals could harbour residual *Plasmodium* parasites or parasite genomic DNA after treatment. The study demonstrates the importance of routine surveillance of asymptomatic malaria using sensitive nucleic acid-based amplification techniques.

## Background

Human malaria remains a global health concern despite the introduction of several control measures over the past decades [1]. In 2020, a global estimate of 241 million malaria cases was recorded, which is comparable to malaria cases reported in 2000 [1]. Among the malaria-vulnerable groups, children under 5 years are the most affected accounting for about 80% of all malaria deaths in 2020 [1]. In sub-Saharan Africa, the region accounting for approximately 95% of total malaria cases [1], four *Plasmodium* species (*Plasmodium falciparum, Plasmodium malariae, Plasmodium ovale* and *Plasmodium vivax*) have been implicated in clinical malaria with *P. falciparum* being the dominant species [2]. The other non-falciparum species have persisted in the background with limited geographical distribution and are usually detected as co-infection with *P. falciparum* [3].

Towards the attainment of malaria elimination and subsequent global eradication, one major setback is the sustained transmission of *Plasmodium* parasites among asymptomatic carriers [3, 4]. These subclinical individuals harbour *Plasmodium* parasites and serve as a reservoir for continuous transmission of the parasites [5]. Most of these subclinical malaria cases are detected as low-density parasitaemia that is often missed or undetected by the commonly used malaria diagnostic tools such as microscopy and antigen-based rapid diagnostic tests (RDTs) [6, 7] due to their limited sensitivity and poor specificity in distinguishing the different *Plasmodium* species [8]. As such, the true extent of asymptomatic malaria infections is usually underestimated in epidemiological studies that involve the use of only microscopy and/or RDTs [9]. Nucleic acid-based amplification tests such as polymerase chain reaction (PCR), on the other hand, provide relatively high analytical performance for species-specific identification of *Plasmodium* species even at low parasite densities [9].

In sub-Saharan Africa, cross-sectional studies in different countries, including Ghana, Uganda, Angola, Nigeria, Malawi and Equatorial Guinea, using PCR-based techniques have reported varying prevalence rates of falciparum and non*-*falciparum species [10–16]. These reports suggest that the transmission dynamics of both falciparum and non-falciparum species vary across different study populations and age groups [3, 17, 18]. In some malaria-endemic settings, changing prevalence rates of the different *Plasmodium* species have been observed following malaria interventions. Notably, a study in Tanzania observed an increasing prevalence of *P. malariae* and *P. ovale* with decreasing prevalence of *P. falciparum* [19]. This observation was also reported in a more recent study in Kenya, where *P. ovale* prevalence increased following a decreased prevalence of *P. falciparum* [20]. In addition, a recent study in Nigeria observed a surprisingly high prevalence of non-falciparum *Plasmodium* species [21]. Other studies have also reported the emergence of non-falciparum *Plasmodium* species after the administration of artemisinin-based combination therapy (ACT) in Ghana [15] and Gabon [22]. These reports highlight the need for routine surveillance of both falciparum and non-falciparum species to improve malaria management.

In Ghana, few PCR-based epidemiological studies have assessed the distribution of falciparum and non-falciparum species among asymptomatic individuals [14, 15, 23–25]. The microscopic prevalence of the genus *Plasmodium* in a cross-sectional study of asymptomatic children living in 30 communities within the Hohoe Municipality of Ghana have been previously reported [26]. In this study, the prevalence rates of *P. falciparum, P. malariae, P. ovale* and *P. vivax* among the same study population were determined using nested PCR, which provides relatively high sensitivity and specificity. In addition to this, the temporal dynamics of *Plasmodium* species and the rate of parasite clearance after ACT were assessed.

## Methods

### Ethical approval

The study was approved by the ethical review committee of the Ghana Health Service (GHS-ERC:14/05/15). In addition, permission was obtained from the Municipal Health directorate, chiefs and elders of the participating communities. Written informed consent was given by the parent or guardian of each participant before enrolment.

### Study area, study design and sample collection

**Fig. 1:**
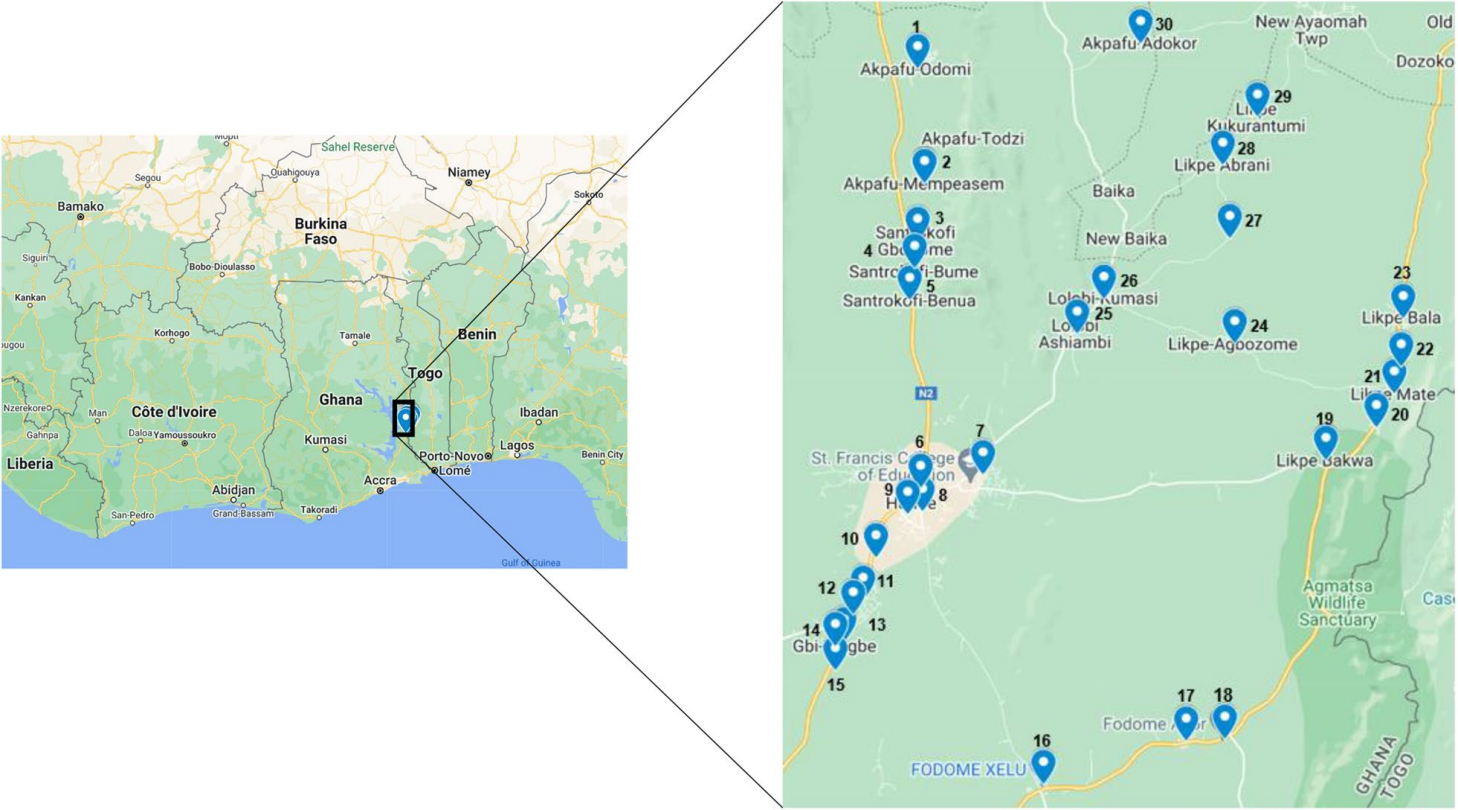
A Google Map of the study sites with insert showing the extended view of the location of the 30 study communities within the Hohoe municipality, Volta Region, Ghana. The 30 study sites have been depicted with blue marks and numbered from 1 to 30, in no particular order

The study was conducted in 30 communities within the Hohoe municipality in the Volta Region of Ghana (Fig. 1). Hohoe is a hyper-endemic region with all-year malaria transmission which peaks in two rainy seasons: one peak in the major rainy season (April to July) and another peak during the minor rainy season (September to November). The entomological inoculation rate (EIR) for the study area is approximately 65 infectious bites per person per year [27]. The current work involved both cross-sectional and longitudinal cohort studies. In the cross-sectional study, a total of 1697 children aged 6–59 months were recruited from the 30 communities in November 2015. Finger-pricked blood samples were collected from the participants for microscopy examination of malaria parasites and dried blood spot (DBS) preparation using Whatman filter paper. A volume of 50 µL of whole blood was used per blood spot. DBS were kept in a desiccant-containing zip-lock bags and stored at room temperature until ready for DNA extraction.

Among the study participants who tested positive (541/1697) for *Plasmodium* species by microscopy, 184 eligible participants were further recruited in the longitudinal cohort study (Cohort 1). The Cohort 1 study participants received a supervised treatment of a 3-day regimen of Artesunate-Amodiaquine (AS + AQ, 25 mg/kg) (IPCA Laboratories, India). The first dose of AS + AQ was given on day 0 by the study team, while subsequent doses were given on day 1 and day 2 by the parent or guardian of each participant. The participants were followed for a period of 70 days, with follow-up on days 7, 14, 28, 42, 56 and 70 after initiating treatment. Finger-pricked blood samples were collected during the follow-up to prepare blood smears for microscopy examination and DBS for PCR analysis.

To assess the rate of malaria parasite clearance, 20 other participants (Cohort 2) from the cross-sectional study who harboured asexual *Plasmodium* parasites, based on microscopy examination, were recruited and treated with a 3-day regimen of AS + AQ (25 mg/kg) under supervision. Baseline samples were taken on day 0 (before treatment). The study participants were then treated on day 0, day 1 and day 2. Additional finger-prick blood samples were collected on days 1, 2 and 3 after initiating treatment to prepare DBS for quantitative PCR (qPCR) assay. These 20 participants (Cohort 2) are independent of the 184 participants (Cohort 1) who were followed over the 70-day period. All DBS were stored at room temperature with desiccant until ready for DNA extraction.

### Microscopy examination of asexual parasites and gametocytes

The details of microscopy preparation and examination have been described previously [27]. Briefly, thick and thin blood smears were prepared at the time of blood sample collection and stained with 10% Giemsa. Asexual parasite count was determined per 200 white blood cells (WBCs), while gametocyte count was estimated per 500 WBCs. Both asexual parasite and gametocyte count per microlitre of blood was determined using the standard count of 8000 leucocytes per microlitre of blood as previously described [28].

### Molecular analysis

#### DNA extraction

Chelex DNA extraction method was used for genomic DNA extraction following previously described protocol [29] with few modifications. Briefly, the DBS were cut and placed in Eppendorf tubes, soaked in 0.5% saponin and incubated overnight at room temperature. The samples were centrifuged and the saponin solution was discarded. The resulting DBS discs were washed three times in excess of 1X PBS. A volume of 200 µL of 2% Chelex-100 solution was added to the DBS discs and incubated at 95ºC for 30 min. The samples were then centrifuged and approximately 150 µL of the resulting supernatant containing the genomic DNA was collected for each sample and stored at − 20 ℃ until ready for PCR assays.

#### Nested PCR (PCR_n_) assays for the detection of *Plasmodium* species

The identification of *Plasmodium* species was determined retrospectively by nested PCR (PCR_n_) using previously reported primer sets targeting the small sub-unit ribosomal genes [30] with few modifications. Briefly, the detection of genus *Plasmodium* (outer PCR) was performed in a total volume of 20 µL containing 1X Standard *Taq* reaction buffer, 200 µM dNTPs, 0.2 µM of each of the forward and the reverse primers, 0.1 µL of *OneTaq* DNA polymerase (BioLabs, New England) and 2 µL of the template genomic DNA (which is approximately 0.7 µL of blood sample). For species-specific analysis, one microlitre of the resulting outer PCR amplicons was used as template in the inner PCR. The cycling conditions for both outer and inner PCR assays included an initial denaturation at 95 ℃ for 5 min followed by 35 cycles of 15 s at 95 ℃, 60 s at 58 ℃ and 90 s at 68 ℃ and a final extension at 68 ℃ for 5 min. A volume of 5 µL of the inner PCR product was separated on 1.5% agarose gel for each sample using the Quick-Load 100 bp DNA Ladder (New England, Biolabs) as the molecular marker. The resulting gels were processed using the Amersham Imager 600 (General Electric Healthcare Life Sciences, Chicago, IL, USA). PCR_n_ assay was performed for the samples obtained from participants in the cross-sectional study (n = 1697) and Cohort 1 (n = 184).

#### Rate of parasite clearance using quantitative PCR (qPCR) analysis

The rate of parasite clearance after ACT for samples from Cohort 2 (n = 20) was determined using SYBR Green-based qPCR assay. This qPCR assay was performed using pan-*Plasmodium* primer set targeting the *Plasmodium* methionine transfer RNA gene which were previously used in a Taqman probe-based qPCR assay developed by Beshir et al. [31]. The detection limit of the SYBR Green-based qPCR assay was not determined in this study. The assays were performed on the Quantstudio5 system (Applied Biosystems). Each of the 20 samples had a day 0 (before treatment) sample and the corresponding day 1, day 2 and day 3 samples after initiating AS + AQ treatment. All reactions were performed in a total volume of 15 µL consisting of 1X Luna qPCR master mix (Bio Labs, New England), 0.2 µM of each primer, and 2 µL of the purified genomic DNA as template. The cycling conditions included an initial denaturation of 5 min at 95 ℃ followed by 40 cycles of 15 s at 95 ℃, and 60 s at 60 ℃. The specificity of the resulting qPCR products was analysed using the melting curves. The mean C_t_-values from technical replicates were used to estimate the relative quantity of parasite genomic DNA. The relative quantity of parasite genomic DNA was estimated from the formula 2^−∆∆ct^ using the day 0 samples (before treatment) as the reference and the human β-tubulin gene for normalization.

#### MSP genotyping

*Plasmodium falciparum* clonal diversity analysis was performed for Cohort 2 samples (day 0, day 1, day 2 and day 3) to investigate possible treatment failure using merozoite-surface protein (MSP) genotyping. MSP1 (K1, MAD20 and RO33) and MSP2 (FC27 and 3D7) allelic families were analysed by nested PCR (PCR_n_) using previously described protocol and primer sets [32]. Briefly, the outer PCR was performed in a 20 µL reaction volume consisting of 1X Standard *Taq* reaction buffer, 150 µM dNTPs, 0.15 µM of each primer, 0.1 µL of *OneTaq* DNA polymerase (New England, BioLabs) and 2 µL of the template DNA. The cycling conditions for the outer PCR included an initial denaturation at 95 ℃ for 5 min followed by 30 cycles of 15 s at 95 ℃, 60 s at 55 ℃ and 90 s at 68 ℃ and a final extension at 68 ℃ for 5 min. The inner PCR consisted of 0.5 µL of the outer PCR product as template DNA. Similar cycling conditions for the outer PCR were used for the inner PCR with varying annealing temperature: K1 and MAD20 at 62 ℃, RO33 at 58 ℃ and MSP2 (FC27 and 3D7) at 57 ℃.

### Statistical analyses

The resulting data were analysed using the IBM SPSS Statistics v26, GraphPad Prism 8.0.2 and Microsoft Excel 2016 Software. Statistical significance for the proportion of positive samples was determined using the Chi-Square test or Fisher’s exact test. Parasite densities were log_10_ transformed and compared across three or more groups using one-way ANOVA test, and where differences were observed, pairwise comparison was conducted using the unpaired t-test. A multivariate analysis was performed to determine the association between age and *Plasmodium* infection, controlling for gender and study site. One-way ANOVA test was used to compare the mean number of clonal infections across the four-time points (days 0, 1, 2, and 3), and differences in the groups were compared by pair-wise analysis using Student’s t-test. All statistical analyses were considered significant for *P* < 0.05.

## Results

### The prevalence of *Plasmodium* species

**Fig. 2:**
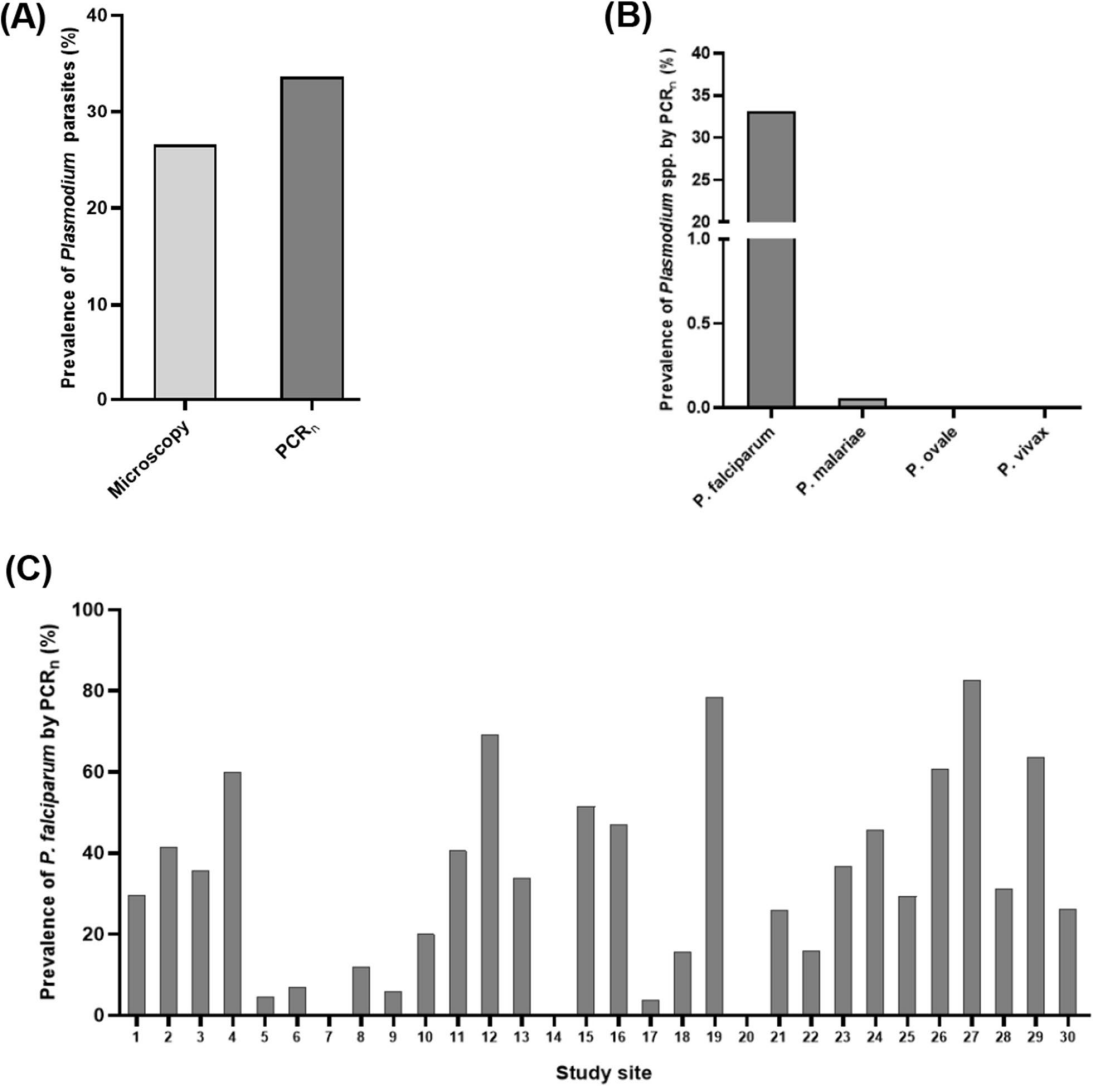
The prevalence rates of *Plasmodium* species among the study participants (n = 1697). **A** The prevalence of *Plasmodium* parasite infection determined by microscopy and nested PCR (PCR_n_). **B** Species-specific distribution of *Plasmodium* parasites by PCR_n_. **C** The distribution of *P. falciparum* across the 30 study sites by nested PCR. The 30 study sites have been numbered 1 to 30, in no particular order, with the corresponding geographical location indicated in Fig. 1

The demographic, and clinical characteristics of the study participants (asymptomatic children, n = 1697) have been described in detail in the previous study [26]. The prevalence rate of *Plasmodium* parasite infection among the study participants in the cross-sectional study as reported in the previous study [26] by microscopy was 26.6% (451/1697) (Fig. 2A). In this study, the total prevalence rate of *Plasmodium* parasite infection by nested PCR (PCR_n_) was 33.6% (571/1697) (Fig. 2A), which was significantly higher compared to that of microscopy (Chi-square, *χ*^*2*^ = 20.16, *P* < 0.0001). Among the microscopy-positive samples, 16.2% (73/451) were negative by PCR_n_. Also, 33.6% (192/571) of the PCR_n_-positive samples were missed by microscopy. For species-specific analysis, the prevalence rates of *P. falciparum*, *P. malariae*, *P. ovale* and *P. vivax* infections by PCR_n_ were 33.6% (570/1697), 0.1% (1/1697), 0.0% (0/1697), and 0.0% (0/1697), respectively (Fig. 2B). Of note, the distribution of *P. falciparum* varied from 0.0% to 82.5% among the 30 study communities (Fig. 2C). Interestingly, no *Plasmodium* species infection was detected among participants from three (3) of the 30 study communities by PCR_n_ (Fig. 2C).

### The temporal dynamics of *Plasmodium* species infection after treatment

**Fig. 3:**
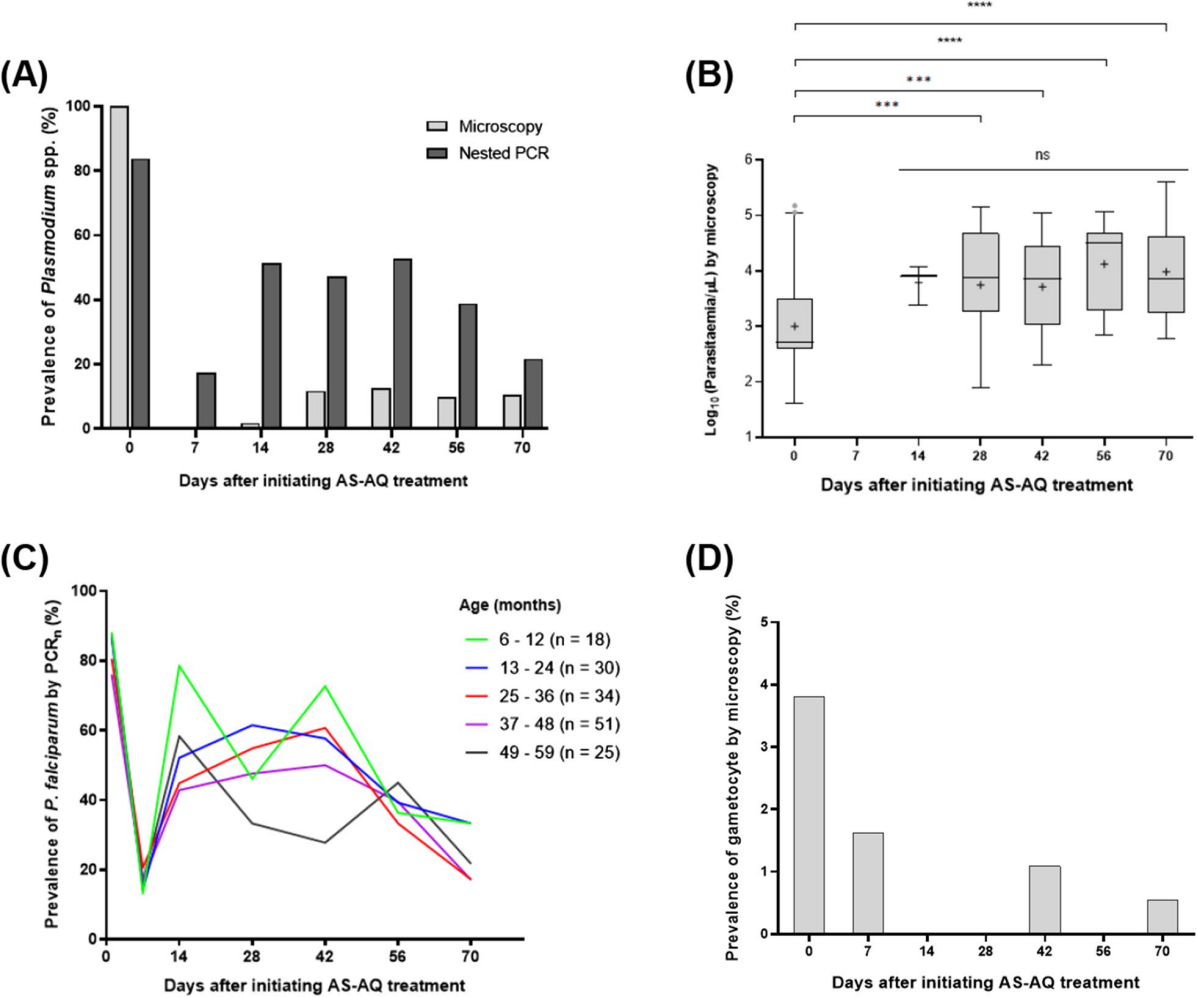
The dynamics of *Plasmodium* parasites after initiating artesunate-amodiaquine (AS-AQ) treatment (n = 184). **A** The overall prevalence of *Plasmodium* parasites over the 70-day period after ACT treatment by microscopy and nested PCR (PCR_n_). **B** Comparison of parasitaemia as determined by microscopy before and after treatment. Data have been presented as box plot and the geometric mean of the parasite load is denoted by the plus symbol ( +). Parasite densities were log_10_ transformed and compared using one-way ANOVA test for three or more groups and unpaired t-test for two groups (^***^P < 0.001, ^****^P < 0.0001 and ns represents not significant). **C** The prevalence of *P. falciparum* by age (months) over 70 days after ACT treatment. **D** The prevalence rate of gametocyte before and after ACT treatment

To assess the dynamics of *Plasmodium* species infection after ACT, a total of 184 participants (Cohort 1) who harboured *Plasmodium* parasites, determined by microscopy, were treated with AS + AQ and followed at weekly or biweekly intervals over a period of 70 days. The prevalence rate of asexual *Plasmodium* parasites by microscopy on days 0, 7, 14, 28, 42, 56 and 70 were 100%, 0.0%, 1.8%, 11.7%, 12.7%, 9.9%, and 10.4%, respectively (Fig. 3A). Using PCR_n_, the prevalence rate of *Plasmodium* parasites on days 0, 7, 14, 28, 42, 56 and 70 were 83.8%, 17.4%, 51.3%, 47.3%, 52.8%, 38.8% and 21.7%, respectively (Fig. 3A). As expected, the observed prevalence rates on all the follow-up days were relatively higher by PCR_n_ compared to the corresponding prevalence by microscopy. There was a substantial reduction in parasite prevalence on day 7 by 100% and 79.2% after treatment by microscopy and PCR_n_, respectively. However, an increase in parasite infection was observed from day 14 to day 42 with a subsequent decline by day 70 by both microscopy and PCR_n_ (Fig. 3A). Based on parasite quantification analysis by microscopy, it was observed that the mean parasitaemia for individuals who harboured parasites was significantly higher on day 28 (*P* = 0.0003), day 42 (*P* = 0.0004), day 56 (*P* < 0.0001) and day 70 (*P* < 0.0001) compared to the mean parasitaemia before treatment (day 0) (Fig. 3B).

The trends in the distribution of *P. falciparum* by PCR_n_ following ACT over the 70-day period were similar when participants were grouped by age (Fig. 3C). However, participants between the ages of 6–12 and 49–59 months had sporadic trends of *P. falciparum* distribution (Fig. 3C).

Also, the odds of *P. falciparum* infection within 28 days after treatment was neither associated with age nor gender (*P* > 0.05 for all comparisons, Table 1). None of the 184 study cohorts was found to harbour non-falciparum species before and after ACT. The prevalence rate of gametocytes as determined by microscopy on days 0, 7, 14, 28, 42, 56 and 70 were 3.8%, 1.6%, 0.0%, 0.0%, 1.1%, 0.0% and 0.5%, respectively (Fig. 3D).

**Table 1:** The odds of *P. falciparum* infection within 28 days after ACT treatment

Characteristics		Odds ratio	95% CI	*P*-value^#^
Gender	Female	1.00		
	Male	0.52	0.26–1.05	0.066
Age (months)	6–12	1.00		
	13–24	0.40	0.10–1.62	0.200
	25–36	0.55	0.17–1.75	0.312
	37–48	0.40	0.13–1.30	0.128
	49–59	0.82	0.30–2.22	0.694

*CI* confidence interval

^#^*P*-value probability value for Pearson Chi-Square test, the female group was used as the reference group for gender analysis, and the age group 6–12 months was used as the reference group for age analysis

### The rate of parasite clearance after ACT

**Fig. 4:**
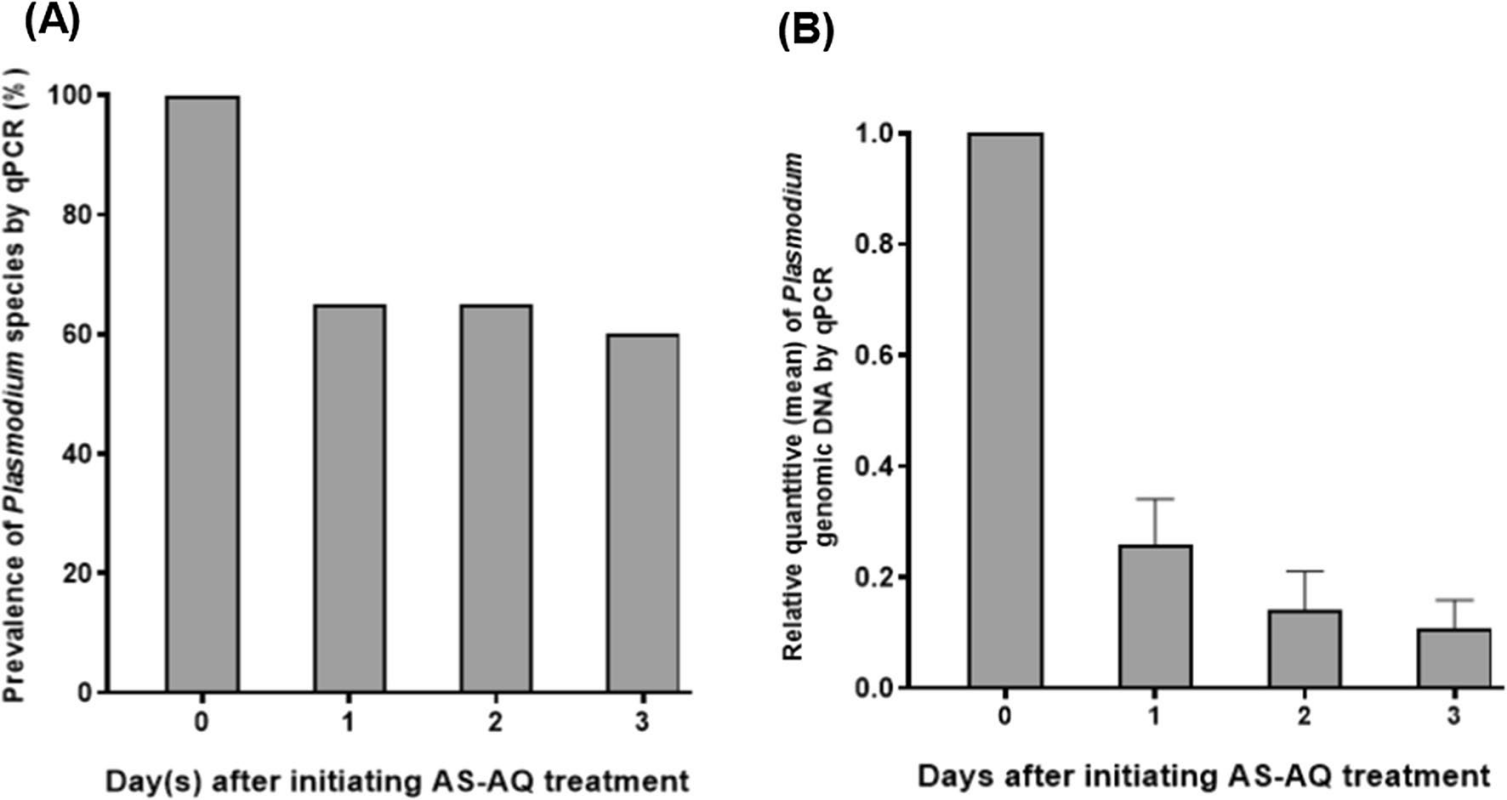
Parasite prevalence before treatment (day 0) and days 1, 2 and 3 after initiating artesunate-amodiaquine (AS-AQ) treatment using SYBR Green-based quantitative real-time PCR (qPCR) (n = 20). **A** Prevalence of *Plasmodium* parasites before treatment (day 0) and after initiating treatment. **B** The relative abundance of parasite genomic DNA before treatment (day 0) and days 1, 2 and 3 after initiating treatment

To determine the rate of parasite clearance, 20 participants (Cohort 2) who harboured *Plasmodium* parasite as determined by microscopy in the cross-sectional study were treated with AS + AQ on days 0, 1 and 2. Samples were obtained from participants on day 0 (before treatment) and days 1, 2 and 3 after initiating treatment. Using qPCR analysis, all the 20 participants were confirmed to harbour *Plasmodium* parasite on day 0 (before treatment). After initiating treatment, the proportion of participants that harboured detectable *Plasmodium* parasite genomic DNA on day 1, day 2 and day 3 were 65.0% (13/20), 65.0% (13/20) and 60.0% (12/20), respectively (Fig. 4A). Compared to day 0 (before treatment), the relative mean quantity of parasite genomic DNA on day 1, day 2, and day 3 were 0.26, 0.14, and 0.11, respectively (Fig. 4B). These indicate percentage reduction in parasite genomic DNA by 74.2%, 85.8% and 88.4% on day 1, day 2 and day 3, respectively. Although there was a decreasing trend in the mean residual parasite genomic DNA levels from day 1 to day 3 (Fig. 4B), the differences were not statistically significant (*P* = 0.283).

### *Plasmodium falciparum* clonal diversity analysis

**Fig. 5:**
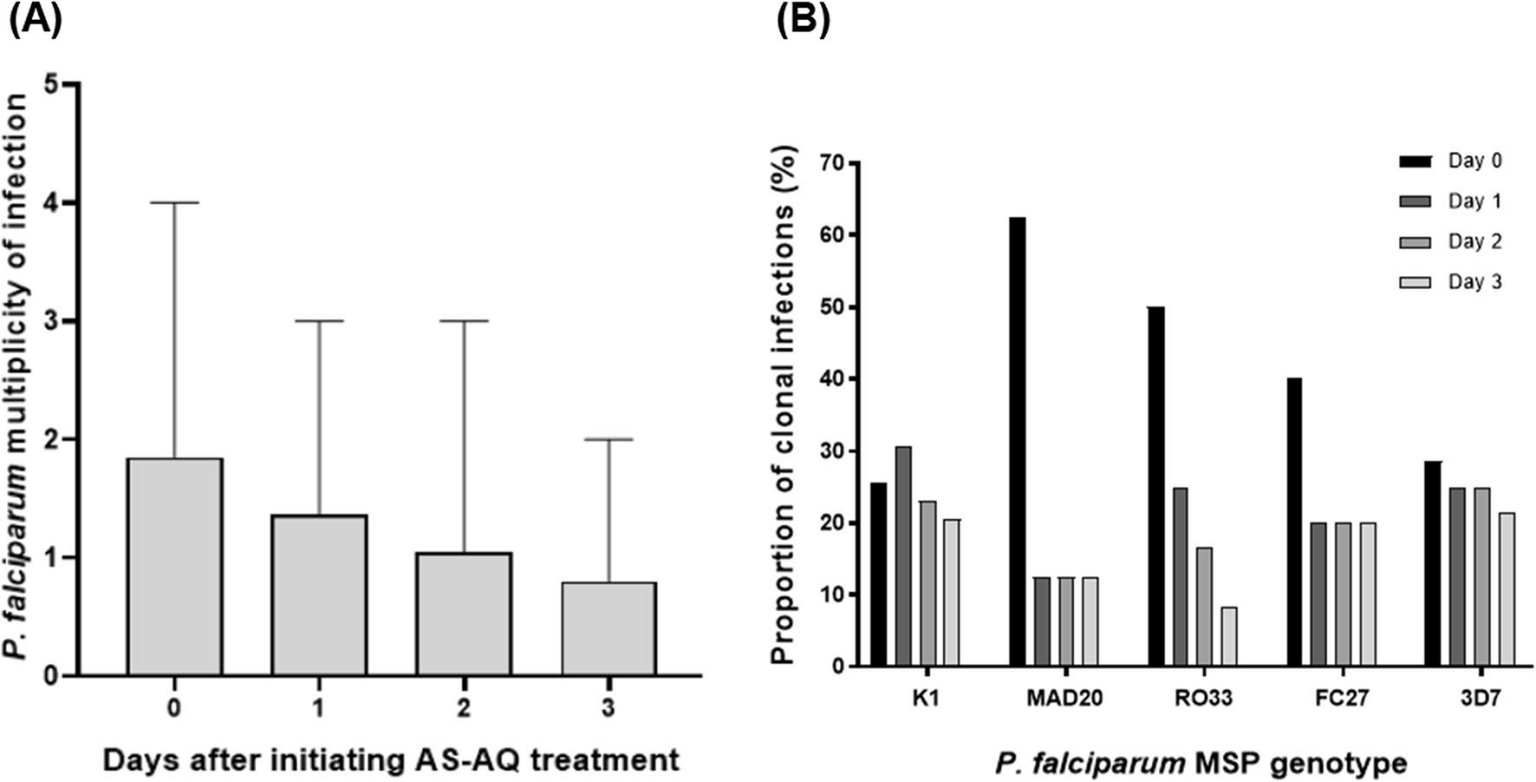
*P. falciparum* clonal infection determined by merozoite-surface protein (MSP) genotyping after initiating artesunate-amodiaquine (AS-AQ) treatment. **A** The average number of *P. falciparum* clones detected per sample before treatment (day 0) and days 1, 2 and 3 after initiating treatment. Error bars represent the standard deviation. **B** The relative proportion of MSP genotypes before treatment (day 0) and days 1, 2 and 3 after initiating treatment

Following the detection of residual parasite genomic DNA on day 3 after initiating treatment, *P. falciparum* parasite clonal diversity (multiplicity of infection) analysis was performed to determine the likelihood of parasite recrudescence or new infection as previously reported [33–38]. Recrudescence was defined as the presence of an allele on day 3 if the same allele was present on either day 0, day 1 or day 2, while new infection was defined as the presence of an allele on day 3 if the same allele was not present on either day 0, day 1 or day 2 [33, 39]. The mean multiplicity of infection on day 0 (before treatment), day 1, day 2 and day 3 were 1.9 [Range: 1–4], 1.4 [Range: 0–3], 1.1 [Range: 0–3], and 0.8 [Range: 0–2], respectively (Fig. 5A). This result indicates a gradual reduction in parasite clonal diversity from day 0 to day 3. As expected, the number of parasite clones for all the five MSP allelic families (K1, MAD20, RO33, FC27 and 3D7) was lowest on day 3 after initiating treatment (Fig. 5B).

On day 0, a total of 32 *P. falciparum* genotypes (K1 = 10, MAD20 = 4, RO33 = 6, FC27 = 4 and 3D7 = 8) were detected among the 20 participants. Of these, 81.2% (26/32) had adequate treatment response, while the remaining 18.8% (6/32) were classified as recrudescence infection. Five of the recrudescence infections [83.3% (5/6)] were of the K1 allelic family. In addition, eleven [11] new infections were detected on day 3, most of which were of the 3D7 allelic family [54.5% (6/11)]. Participants with residual parasite genomic DNA on day 3 resulting from either recrudescence and/or new infection generally had higher multiplicity of infection on day 0 than those with adequate treatment response, however, the difference was not statistically significant (*P* = 0.87).

## Discussion

In this study, a cross-sectional analysis was first performed to determine the prevalence of *Plasmodium* species by nested PCR. As expected, the prevalence rate of *Plasmodium* parasites by nested PCR was significantly higher than that of microscopy. Compared to nested PCR, 33.6% of the PCR positives were misdiagnosed by microscopy. *Plasmodium falciparum* was the most prevalent (33.6%) *Plasmodium* species among the study participants. Following ACT, a significant proportion of the participants was found to harbour *Plasmodium* parasites, with a fluctuating prevalence rate that increased from day 14 to day 42 and a subsequent decline by day 70. Interestingly, the mean parasitaemia determined by microscopy from day 28 to day 70 was significantly higher compared to pre-treatment. Further analysis of the rate of parasite clearance indicates the presence of residual parasites or parasite genomic DNA, with a decline in clonal diversity from day 0 (before treatment) to day 3 after initiating ACT. These observations highlight the importance of routine surveillance of *Plasmodium* species using nucleic acid-based amplification assays to obtain reliable data that would inform the implementation of effective control measures and interventions [40].

This present study observed a considerable high number of PCR_n_-positive samples [33.6% (192/571)] that were undetected by microscopy and microscopy-positive samples [16.2% (73/451)] that were also missed by PCR_n_. These observed discrepancies may be explained by factors including the lower detection limit of PCR compared to microscopy [9], and the technical expertise and high quality control required for microscopy [41–44]. This means that even a slight fall in standards can lead to false positive or false negative results [43, 44]. For species-specific analysis, the observed prevalence rate of *P. falciparum* (33.6%) in this study is comparable to reports from previous studies involving asymptomatic children under 5 years in Ghana (36.8%) (25), Burkina Faso (38.2%) [45], and Nigeria (29.0%) [46]. However, other studies in Ghana reported higher prevalence among older children in the Ashanti region (66%) [15], and the Eastern region (63.8%) [47]. The differences in the observed prevalence may be due to age-dependant immunity since the pattern in the distribution of *Plasmodium* species infection has been associated with age [48–50]. In addition, variation in malaria transmission intensity across the study sites could also account for the observed differences in the prevalence of *P. falciparum* [10, 51, 52].

The current study also observed a very low carriage of non-falciparum species (< 0.1%) among the study participants. These frequencies are within the range of the estimated national prevalence of < 10% for *P. malariae,* < 2% for *P. ovale* and 0.0% for *P. vivax* in Ghana [53]. This observation corroborates findings in the Volta region where this study was performed [54] and in other studies in Ghana [16], Burkina Faso [55], Zambia [56] and Uganda [57]. On the contrary, other studies conducted elsewhere in Ghana have reported higher frequencies of non-falciparum species among asymptomatic children [14, 15]. Also, a more recent study in Nigeria among adolescents aged 10–19 years observed an unexpectedly high prevalence of 66.4% and 30.5% for *P. malariae* and *P. ovale*, respectively [22]. The differences in the dynamics of non-falciparum species across different sites could be due to the variations in population characteristics, geographic and seasonal transmission [3, 18]. In addition, the use of other PCR-based methods including cooperative primer-based assays [17] and primers targeting mitochondria DNA [58] may be suitable or optimal for the detection of non-falciparum malaria parasites since they are usually detected as low-density infection.

Using PCR analysis, we observed a significant proportion of the study participants harbouring detectable parasite genomic DNA after ACT for both the 70-day (Cohort 1) and the 3-day (Cohort 2) follow-up participants. Previous studies have also reported sub-patent parasite loads after ACT in Ghana [15], Kenya [33], Uganda [35], Angola [36] and Tanzania [38]. Another study in Zanzibar associated sub-patent parasite that was observed post-treatment with factors including parasite density at enrolment, age, baseline temperature and haemoglobin levels [59]. Due to the limited sample size (n = 20, Cohort 2) of these treated participants in the current study, these previously reported factors were not investigated**.** It is important to highlight that a 3-day regimen of ACT is generally known to rapidly clear asexual malaria parasites and is also expected to clear both new or developing *Plasmodium* infections [60–62]. Based on the microscopy analysis in this study, one possible explanation for the observed parasites or parasite genomic DNA during the follow-up could be due to the presence of gametocytes that were not cleared after treatment as reported in previous studies [15, 33]. In addition, there is the possibility of parasite re-infection or recrudescence after treatment [33–38]. Also, circulating parasite genomic DNA released from dead parasites could account for the observed parasite genomic DNA detected during and after treatment [63, 64].

The study further performed parasite clonal analysis using MSP genotyping to investigate the possibility of parasite re-infection or recrudescence. It is important to highlight that MSP genotyping was done for only the 3-day follow-up participants (Cohort 2) and not the 70-day follow-up participants (Cohort 1) due to financial constraints. Based on the parasite clonal diversity analysis, it was observed that the residual parasites or parasite genomic DNA detected on day 3 after initiating treatment was due to both recrudescence and new infections. This observation is in line with previous studies in Kenya [33] and Tanzania [38], where recurrent infections were associated with recrudescence and new infections. Of note, there is the possibility of underestimating recrudescent infections due to undetected parasite clones in the pre-treatment sample which become detectable after drug exposure reduced parasite density, as previously noted [33]. Over-estimating the number of recrudescent infections is theoretically possible, especially within a specific geographic location where some genetic relatedness is possible, but this is far less likely in high transmission, and high complexity settings such as Hohoe [65–68]. In addition, circulating genomic DNA from dead parasites could potentially lead to over-estimation of parasite recrudescence or re-infections [62, 63]. Other possible factors that may explain the observed “new” infections have been described in details elsewhere [33]. A further study is recommended to accurately determine the source of the residual parasites or genomic DNA after treatment.

Taken together, the results presented here suggest a high transmission intensity at the study site amidst malaria control measures and interventions [27]. These observations have been associated with resistance to anti-malarial drugs such as chloroquine and sulfadoxine-pyrimethamine [68, 69]. This calls for the routine application of sensitive molecular-based tools for monitoring anti-malarial drug efficacy.

## Conclusion

The study demonstrates the importance of routine community-based surveillance of *Plasmodium* species using sensitive nucleic acid-based amplification assays. As expected, *P. falciparum* was the dominant *Plasmodium* species among the study participants. The residual parasites or parasite genomic DNA observed after treatment necessitates further studies among a larger study population to properly establish the efficacy of ACT in Ghana. In addition, the use of RNA-based detection methods, parasite culturing and in vitro drug assays will be necessary to accurately assess the efficacy of ACT against both asexual and sexual stage parasites in human blood.

## Data Availability

All data presented in this manuscript are available on reasonable request from the corresponding author.
